# Microbiology of Chronic Suppurative Otitis Media: An update from a Tertiary Care Hospital in Bangladesh

**DOI:** 10.12669/pjms.37.3.3942

**Published:** 2021

**Authors:** Mst. Romena Khatun, Kh. Md. Faisal Alam, Mahmuda Naznin, Md. Abdus Salam

**Affiliations:** 1 Mst. Romena Khatun, M, Phil. Lecturer, Department of Virology, Rajshahi Medical College, Rajshahi-6000, Bangladesh; 2 Kh. Md. Faisal Alam, PhD. Associate Professor Department of Microbiology, Rajshahi Medical College, Rajshahi-6000, Bangladesh; 3 Mahmuda Naznin, M. Phil. Assistant Professor, Department of Microbiology, Rajshahi Medical College, Rajshahi-6000, Bangladesh; 4 Prof. Md. Abdus Salam, PhD, FRCP (UK). Department of Basic Medical Sciences, Kulliyyah of Medicine, International Islamic University Malaysia, 25200 Kuantan, Pahang, Malaysia

**Keywords:** CSOM, Antibiogram, Multidrug-resistance, Tertiary care hospital, Bangladesh

## Abstract

**Objectives:**

Chronic suppurative otitis media is a major cause of acquired hearing impairment, especially in children of developing countries. The study sought to explore the bacteriological profile and their antimicrobial susceptibility among patients of chronic suppurative otitis media from a tertiary care hospital in Bangladesh.

**Methods:**

A cross sectional microbiological study was conducted at the Department of Microbiology, Rajshahi Medical College, Bangladesh from January to December 2019. Aural swabs were collected aseptically from clinically suspected patients irrespective of age and gender attending the ear, nose and throat outpatient department of Rajshahi Medical College Hospital. Aerobic bacterial culture was done and isolates were identified through standard bacteriological identification scheme. Antimicrobial susceptibility testing of isolates was done by modified Kirby-Bauer disk diffusion method following Clinical and Laboratory Standards Institute guidelines.

**Results:**

Of 96 swabs, culture yielded a total of 73 bacterial isolates from 68(70.8%) culture-positive plates including 63 (65.6%) unimicrobial and 5 (5.2%) polymicrobial (mixed growth of a pair of bacteria) growths. Frequency distribution revealed, 40(55%) gram-negative and 33(45%) gram-positive bacteria with Staphylococcus aureus was the leading isolate (37%) followed by Pseudomonas aeruginosa (31.5%), Escherichia coli (13.7%), coagulase-negative Staphylococcus (8.2%), Klebsiella pneumoniae (5.5%) and Proteus spp. (4.1%). Gram-positive bacteria were found to be highly susceptible (100%) to Linezolid and Vancomycin followed by Imipenem (83 to 96.3%), while moderate to high resistance (44 to 67%) was observed against Ciprofloxacin, Ceftriaxone, Ceftazidime, Amoxicillin/Clavulanate and Clindamycin. For gram-negative bacteria, susceptibility ranged from 67 to 100% to Imipenem, 67 to 96% to Piperacillin/Tazobactam and 67 to 83% to Gentamicin, while moderate to high resistance (50 to 75%) was observed against Ciprofloxacin, Ceftriaxone, Ceftazidime and Amoxicillin/Clavulanate.

**Conclusion:**

Moderate to high level of multidrug-resistance especially to 3^rd^ generation cephalosporins, Ciprofloxacin and Amoxicillin/Clavulanate is an alarming situation. It warns reinforcement of judicious antibiotic prescription and introduction of antibiotic stewardship program in the tertiary care hospitals.

**List of abbreviations:**
CSOM: chronic suppurative otitis media;
MDR: multidrug-resistance;
RMCH: Rajshahi Medical College Hospital;
CLSI: Clinical and Laboratory Standards Institute;
ATCC: American Type Culture Collection;
MRSA: Methicillin-resistant *Staphylococcus aureus*;
MSSA: Methicillin-susceptible *Staphylococcus aureus*;
CoNS: coagulase-negative *Staphylococcus*;
AST: antimicrobial susceptibility testing;
AMR: antimicrobial resistance;
ASP: antibiotic stewardship program.

## INTRODUCTION

Chronic suppurative otitis media (CSOM) is still a significant health problem in resource-limited settings and is an important cause of preventable hearing loss, particularly in the children. Global burden of illness from CSOM estimates 65-330 million individuals with over 90% is born by countries in the Southeast Asia, Western Pacific regions and Africa. Prolonged and often recurring bacterial infection of the ear cleft involving Eustachian tube, middle ear and mastoid accompanied by perforation of the tympanic membrane leading to discharge (otorrhoea) lasting more than two weeks refers to CSOM according to the World Health Organization, although otolaryngologists tend to adopt a longer duration usually more than three months.^1^

Incidence of this disease is higher in developing countries especially in low socioeconomic groups affecting seriously the quality of life. Risk factors include frequent episodes of acute otitis media, other respiratory tract infections and traumatic tympanic rupture as well as factors underlying the resource-limited living conditions such as overcrowding, poor nutrition and hygiene and chronic infectious diseases.^2^,^3^ In Bangladesh, the prevalence of CSOM was found to be 7.4 to 39.5% and it was 7.8 to 16% in India reported in recent studies.^4^-^6^ Dispersal of bacteria from a biofilm in the middle ear could serve as a potential reservoir and explain the recurrent and chronic nature of CSOM with reduce efficacy of antibiotic treatment.^7^

Microbiota of CSOM include aerobes, anaerobes and fungi as potential pathogens though its reported profile and frequency differ based on patient’s age, geography and the presence of complications like cholesteatoma.^8^ Frequently isolated aerobic bacteria include *P. aeruginosa, S. aureus, E. coli, S. pyogenes, K. pneumoniae* and *Proteus* spp. or mixed infections. While common anaerobes are *Bacteroides*, *Peptostreptococcus* and *Propionibacterium*.^2^,^4^,^9^ Like other bacterial infections, there is a growing concern of multidrug-resistant (MDR) bacteria in CSOM. High level of antimicrobial resistance against commonly used antibiotics like Ampicillin, Amoxicillin, Cotrimoxazole, Amoxicillin/Clavulanate, Cephalosporins, Quinolones and Macrolides has been a challenge leading to unsatisfactory treatment outcomes in the recent years.^2^,^5^,^10^,^11^

Continuous and periodic antibiogram in CSOM is always a pressing need to guide treatment and to provide records for future reference in view of changing antimicrobial susceptibility. Literature review indicates that only a few microbiological investigations of CSOM so far have been carried out in Bangladesh and still there is lack of updates in information. The present study was aimed to explore the current trend in bacteriological profile and their antimicrobial susceptibility among CSOM patients from a tertiary care hospital in Bangladesh.

## METHODS

The research protocol was approved by the ‘Ethical Review Committee’ of Rajshahi Medical College, Bangladesh and informed written consent/assent was taken from patient (Ref: RMC/ERC/2017-2019/177/189, Dated: 07-12-2019). This cross-sectional microbiological investigation used purposive sampling technique, conducted from January to December, 2019 among ninety-six (96) clinically suspected patients with CSOM of different age and gender. Patients were recruited from the ear, nose and throat (ENT) outpatient department of Rajshahi Medical College Hospital (RMCH), a 1000-bed tertiary care teaching hospital in the Northern part of Bangladesh.

### Inclusion Criteria:

Persistent or intermittent unilateral or bilateral ear discharge through a perforated tympanic membrane with a duration of over 12 weeks who were not on local or systemic antibiotic therapy for seven days prior to sample collection.

### Exclusion Criteria:

Patients with acute suppurative otitis media, chronic otitis media with effusion, otitis media with concomitant otitis externa and unwilling for voluntary participation.

### Collection of Swabs:

Sterile cotton tipped swab stick was used to collect two aural swabs aseptically from discharging ear of each patient; one for microscopy and the other for bacterial culture. After cleaning the auditory canal with 70% ethanol, under good light and guidance of a sterile aural speculum, swab stick was passed into the middle ear to collect pus avoiding contact with surrounding skin. Swabs were put in the sterile tubes and capped with sterile cotton plugs before transferring to the Microbiology laboratory as soon as possible.

### Microscopical Examination:

A thin uniform smear was prepared with each sample and was air dried before fixing by flaming. Gram staining was done to the fixed smear to see gram reaction, morphology and arrangement of bacteria under microscope using oil immersion (x100) objective.

### Culture & Identification of Bacteria:

All specimens were inoculated into nutrient agar, blood agar and MacConkey’s agar media (Oxoid, UK) and incubated aerobically at 37°C for 18-20 h. Identification of bacteria was based on microscopy (gram reaction, shape, arrangement) and colony characteristics (colony morphology, haemolysis on blood agar, changes in the physical appearance of the differential media). Gram-positive isolates were tested for catalase and coagulase while gram-negative isolates were tested for oxidase, citrate utilization, motility indole urease and triple sugar iron tests.^12^

### Antimicrobial Susceptibility Testing (AST):

Antibiotic susceptibility of isolated bacterial pathogens was performed using modified Kirby Bauer disc diffusion method according to the guidelines of the Clinical and Laboratory Standard Institute (CLSI). A colony suspension with concentration equivalent to 0.5 McFarland solution was prepared for each isolate and inoculated into Mueller-Hinton agar (Oxoid, UK). Selected commercially available antibiotic discs (Oxoid, UK) were placed onto the media and incubated at 37°C for 24 hour. Results were noted as *resistant* and *susceptible* according to CLSI guideline.^13^

Gram-positive bacteria were tested against Amoxicillin/Clavulanate (20/10 μg), Cefoxitin (30 μg), Ceftazidime (30 μg), Ceftriaxone (30 μg), Clindamycin (02 μg), Ciprofloxacin (05 μg), Gentamicin (10 μg), Imipenem (10 μg), Linezolid (30 μg) and Vancomycin (30 μg). Gram-negative bacteria were tested against Amoxicillin/Clavulanate (20/10 μg) Ceftazidime (30 μg), Ceftriaxone (30 μg), Cefepime (30 μg), Ciprofloxacin (05 μg), Gentamicin (10 μg), Imipenem (10 μg) and Piperacillin/Tazobactam (100/10 μg).

*Escherichia coli* ATCC 25922 and *Staphylococcus aureus* ATCC 25923 were used as control strains for AST.^13^ Resistance to at least one agent in three or more antimicrobial categories was defined as MDR.^14^

### Data Collection and Statistical Analysis:

A partially structured pre-tested questionnaire was used for recording patient’s demographic, clinical and microbiological data. Nominal variables were shown as number of cases (*n*) and percentage (%). Descriptive statistical methods in SPSS (version 21.0 for Windows, SPSS® Inc., Chicago, IL) were applied for data analysis.

## RESULTS

**Fig.1:**
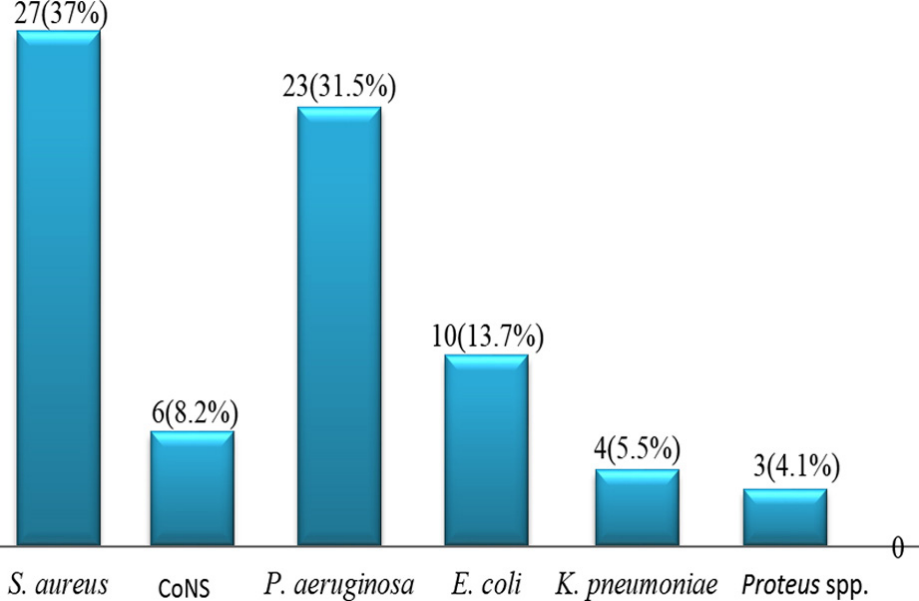
Frequency distribution of bacteria isolated from CSOM patients (n=73).

Age ranged from <10 to 70 years with a mean age of 26.9 ± 15.1 years among culture positive CSOM patients. Majority 61 (63.5%) cases were from rural locality and there was female preponderance with a male to female ratio of 1:1.2. Out of 96 cases, culture yielded a total of 73 bacterial isolates from 68 (70.8%) culture-positive samples including 63 (65.6%) unimicrobial and 5 (5.2%) polymicrobial (mixed growth of a pair of bacteria- 5x2=10) growths. For bacterial isolates, 40 (55%) were gram-negative and 33 (45%) were gram-positive with *S. aureus* was the leading isolate (37%) followed by *P. aeruginosa* (31.5%), *E. coli* (13.7%), coagulase-negative *Staphylococcus* (CoNS) (8.2%), *K. pneumoniae* (5.5%) and *Proteus* spp. (4.1%) (Fig.1). Of 27 isolates of *S. aureus*, 23 (85.2%) were Methicillin sensitive *Staphylococcus aureus* (MSSA) and 04 (14.8%) were Methicillin resistant *Staphylococcus aureus* (MRSA). The combination of bacterium in polymicrobial growths and their frequency distribution are shown in Table-I. Combination of gram-positive and gram-negative bacterium was observed with predominance of *S. aureus*.

**Table I:** Pattern of polymicrobial growths from CSOM patients.

Bacterial combination	n (%)
S. aureus + E. coli	2 (2.9)
S. aureus + K. pneumoniae	2 (2.9)
P. aeruginosa + CoNS	1 (1.5)

Total	5 (7.3)

Regarding antimicrobial susceptibility, gram-positive bacteria were found to be 100% susceptible to Linezolid and Vancomycin followed by Imipenem (83 to 96.3%). Moderate to high resistance ranging from 44 to 67% was observed for gram-positive bacteria against Ciprofloxacin (44%), Ceftriaxone (48%), Ceftazidime (63%), Amoxicillin/Clavulanate (63%) and Clindamycin (67%). For gram-negative bacteria, susceptibility ranged from 67 to 100% to Imipenem, 67 to 96% to Piperacillin/Tazobactam and 67 to 83% to Gentamicin. While moderate to high resistance ranging from 50 to 75% was observed against Ciprofloxacin (50%), Ceftriaxone (67%), Ceftazidime (67%) and Amoxicillin/Clavulanate (75%) (Table-II).

**Table II:** Antimicrobial susceptibility pattern of bacteria from CSOM patients (n=73).

Bacteria	n (%) of bacteria susceptible towards different antibiotics											

	AMC	CTX	CAZ	CRO	CPM	IMP	DA	VAN	LZ	CIP	GN	PTZ
***Gram-positive***											
S. aureus	10 (37)	23 (85.2)	10 (37)	14 (52)	-	26 (96.3)	09 (33.3)	27 (100)	27 (100)	15 (55.5)	22 (81.4)	-
CoNS	3 (50)	6 (100)	3 (50)	4 (66.7)	-	5 (83)	3 (50)	6 (100)	6 (100)	4 (66.7)	5 (83)	-
***Gram-negative***											
P. aeruginosa	6 (26.1)	-	10 (43.5)	11 (47.8)	18 (78.2)	22 (95.6)	-	-	-	15 (65.2)	19 (82.6)	22 (95.6)
E. coli	3 (30)	-	6 (60)	5 (50)	7 (70)	8 (80)	-	-	-	7 (70)	7 (70)	9 (90)
K. pneumoniae	1 (25)	-	2 (50)	2 (50)	3 (75)	4 (100)	-	-	-	2 (50)	3 (75)	3 (75)
Proteus spp.	1 (33.3)	-	1 (33.3)	1 (33.3)	2 (66.7)	2 (66.7)	-	-	-	2 (66.7)	2 (66.7)	2 (66.7)

**AMC**- Amoxicillin/Clavulanate, **CTX**-Cefoxitin, **CAZ**-Ceftazidime, **CRO**-Ceftriaxone, **CPM**-Cefepime, **IMP**-Imipenem, **VAN**-Vancomycin, **DA**-Clindamycin, **LZ**-Linezolid, **CIP**-Ciprofloxacin, **GN**-Gentamicin, **PTZ**-Piperacillin/Tazobactam.

## DISCUSSION

Emergence of resistance to commonly used antibiotics is a great concern worldwide causing treatment failure in different infections including CSOM.^15^ Regarding demographic attributes, we noted slightly female preponderance, rural predominance and younger age among CSOM patients and similar trends were also observed by others.^4^,^9^,^16^ These attributes are inherent for CSOM, mostly prevalent in the developing countries. Gram-negative bacteria accounted for higher isolation rate than gram-positive (55% vs 45%), which corroborates with many other studies.^5^,^6^,^17^,^18^ The reason for higher prevalence could be the chronic nature of infection where gram-negative bacteria from external sources get access into the auditory canal, suppressing the gram-positive normal flora and eventually become predominant. The leading bacterial pathogen was *S. aureus* including 15% MRSA that corroborates well with a few studies.^2^,^5^,^19^ The association of *S. aureus* in middle ear infections can be attributed to its ubiquitous nature and high carriage of MRSA in the external auditory canal and upper respiratory tract. We noted *P. aeruginosa* to be the second most prevalent isolate followed by *E. coli*, CoNS, *K. pneumoniae* and *Proteus* spp., which is in agreement with others.^2^,^7^ Although *P. aeruginosa* has been reported to be the leading cause of CSOM followed by *S. aureus* by many investigators but variation in bacterial profile and frequency is well documented.^10^,^11^,^20^ The conducive environment of middle ear cavity may favour the growth of *P. aeruginosa* but etiological agents of CSOM differs substantially with regards to time and place and also factors like variation in climate, socioeconomic status and patient’s self-hygiene play important role.^11^ We observed polymicrobial infections in 7.3% cases, which is a very common occurrence in CSOM and many studies have reported mixed pathogens including bacteria and fungus.^5^,^8^,^9^ Polymicrobial growths may be well explained by the fact that the perforated ear drum facilitates coliforms such as *E. coli*, *K. pneumoniae* and *P. aeruginosa* to establish infection in wet and poor hygienic environment.

Linezolid and Vancomycin were found 100% efficacious against gram-positive bacteria followed by Imipenem (83 to 96.3%) and Gentamicin (81.4 to 83%) and similar efficacy has also been reported by others.^2^,^17^,^18^,^21^ Until now, Vancomycin, Linezolid and Imipenem are among less frequently prescribed antibiotics for CSOM, still preserving their excellent efficacy. This conservation should be continued to combat emergence of resistance. Clindamycin is usually an alternative choice for Penicillin-sensitive individuals was found to be poorly efficacious with 50 to 67% resistance against gram-positive bacteria and this observation is in accordance with another study.^22^ Most effective antibiotics for gram-negative bacteria was Imipenem, followed by Piperacillin/Tazobactam and Gentamicin showing their susceptibility ranging from 67 to 100%. Moderate to high resistance (50 to 75%) was observed against Ciprofloxacin, Ceftriaxone, Ceftazidime and Amoxicillin/Clavulanate and our results corroborate with others regarding gram-negative bacterial susceptibility.^17^,^18^,^20^,^23^ Although Cefepime, the 4^th^ generation cephalosporin turned out to be good with 67 to 78% susceptibility but 3^rd^ generation cephalosporins like Ceftazidime and Ceftriaxone were found unsatisfactory. Likewise, Ciprofloxacin, a frequently used fluroquinolone was also found to be less efficacious with resistance ranging from 35 to 50% against both gram-positive and negative isolates, which coincides to some but contradicts others.^6^,^11^,^19^ Further, both gram-positive and gram-negative isolates showed 50 to 75% resistance against Amoxicillin/Clavulanate, which has long been used as the empirical drug of choice for CSOM. In a systematic review and meta-analysis, Tesfa *et al*. reported high level of resistance against Ampicillin, Amoxicillin/Clavulanate, Cotrimoxazole, Amoxicillin, and Cefuroxime and our finding is in concordance with this report and also in agreement with another recent study.^10^,^20^ Moderate to high resistance observed against different classes of antibiotics indicates prevalence of MDR bacteria and is a matter of great concern because antibiotics from these classes are being prescribed frequently for empirical treatment of CSOM. Unethical prescription of antibiotic for trivial causes, easy availability over the counter, lack of culture facilities and absence of good governess are all contributing factors in the development of high antimicrobial resistance (AMR) in the developing countries including Bangladesh. Conservation of antibiotic must be optimized and implemented through awareness of current situation of AMR and educational programs targeting both antimicrobial prescribers and consumers. Further, employment of antibiotic stewardship program (ASP) to optimize both inpatient and ambulatory antimicrobial prescription practices in tertiary hospitals is highly recommended for an overall effectiveness of conservation programs.

### Limitations of the study:

Sample size was relatively small and anaerobic bacteria and fungus could not be isolated. Also, genotypic confirmation of resistant bacteria could not be done.

## CONCLUSIONS

Linezolid, Vancomycin and Imipenem have been found to be highly effective drugs for gram-positive bacteria while Imipenem, Piperacillin/Tazobactam and Gentamicin were better choice for gram-negative bacteria causing CSOM. Moderate to high level of multidrug-resistance especially to 3^rd^ generation cephalosporins, Ciprofloxacin and Amoxicillin/Clavulanate is an alarming situation. Continuous and periodic evaluation of microbiological profile and antimicrobial sensitivity is imperative for optimum treatment and to combat antimicrobial resistance in CSOM. Our findings warn seriously towards rational use of empirical antibiotics for CSOM and introduction of antibiotic stewardship program in the tertiary care hospitals.

### Authors’ contributions:

**MRK, KFA & MAS:** conceived, designed and manuscript writing.

**MRK & MN:** laboratory works, data collection and analysis.

**MAS:** Revised for intellectual contents and responsible for accuracy and integrity of the work.

All authors have read and approved the final form of the manuscript.
